# Effect of Citrulline and Leucine Intake with Exercises on Body Composition, Physical Activity, and Amino Acid Concentration in Older Women: A Randomized Double-Blind Placebo-Controlled Study

**DOI:** 10.3390/foods10123117

**Published:** 2021-12-15

**Authors:** Mijin Kim, Hiroko Isoda, Tomohiro Okura

**Affiliations:** 1 R&D Center for Tailor-Made QOL, University of Tsukuba, Tsukuba 305-8550, Japan; isoda.hiroko.ga@u.tsukuba.ac.jp (H.I.); okura.tomohiro.gp@u.tsukuba.ac.jp (T.O.); 2 Alliance for Research on the Mediterranean and North Africa, University of Tsukuba, Tsukuba 305-8572, Japan; 3 Faculty of Life and Environmental Sciences, University of Tsukuba, Tsukuba 305-8572, Japan; 4 Faculty of Health and Sport Science, University of Tsukuba, Tsukuba 305-8572, Japan

**Keywords:** leucine, citrulline, body composition, physical activity, aging, low BMI, sarcopenia

## Abstract

The combined intake of citrulline (CIT) and leucine (LEU) can stimulate protein synthesis. Therefore, this study aimed to investigate the effect of combined intake of CIT and LEU accompanied by exercise for 20 weeks on body composition, physical activity (PA), and amino acid concentrations in older Japanese women with low body mass index (BMI) (16 to 21 kg/m^2^) using a randomized, double-blind, placebo-controlled design. The supplement was administered twice a day for 20 weeks (Ex (exercise) + CIT·LEU group, *n* = 10: mainly 0.8 g CIT and 1.6 g LEU; Ex + Placebo group, *n* = 13: mainly 3.5 g carbohydrate). Additionally, both groups exercised (weight-bearing exercise, square stepping exercise) once a week for 75 min. Body composition, PA, and amino acid concentrations in the plasma were measured. Body weight, BMI, body mass, household PA, total PA, and phenylalanine significantly increased in the Ex + CIT·LEU group (*p* < 0.05) post intervention. This study suggests that the combined intake of CIT and LEU accompanied by exercise can improve body weight, BMI, body mass, and PA in older women with low BMI, which may prevent sarcopenia and frailty.

## 1. Introduction

Aging strongly affects the body’s composition, particularly bone mass, muscle mass, and estimated somatic cell mass from intracellular fluid volume [1]. Muscle mass begins to decrease at age 25, with the decrease accelerating at age 50, and by 80 years has decreased by an average of 40% compared with age 20 [2]. Loss of body weight and muscle mass with aging, which is accelerated because of nutritional deficiencies (dietary protein), leads to a decrease in physical function and physical activity [3]. The low BMI of older individuals is strongly associated with sarcopenia, i.e., age-related loss of skeletal muscle mass, strength, and function [4]. The prevalence of sarcopenia in Japan was higher in women (16.7%) than men (11.5%) [5]. Sarcopenia has been associated with disabilities in the performance of instrumental activities of daily living, poor physical functioning, low nutritional intake, and mortality [6]. The combined intervention of amino acid intake and exercise has been shown to increase muscle protein synthesis (MPS) [7] and leg muscle mass [8] in older adults. Weight-bearing exercise (WBE), which can be easily performed without equipment at any time, has been shown to improve bone mineral density [9] and promote muscle activation [10]. Moreover, square stepping exercise (SSE), an aerobic exercise, has been reported to improve both lower-extremity function [11] and prevent falls [12] in older adults. Furthermore, combined intervention of nutrition and exercise has shown more effectiveness in the improvement of the body composition of underweight older adults compared with the single use of either one.

Leucine (LEU) is a well-known essential amino acid for protein metabolism and is one of the three proteinogenic branched-chain amino acids along with isoleucine and valine [13]; a unique characteristic of LEU is the stimulation of protein synthesis. Through the activation of the mammalian target of rapamycin (mTOR), LEU regulates the signaling pathway of insulin PI 3-kinase and stimulates the translational control of protein synthesis [14]. In addition, citrulline (CIT), a non-protein amino acid, is a potent endogenous precursor of arginine. Direct intake of arginine can cause gastrointestinal distress; however, intake of CIT can reduce gastrointestinal distress and promote arginine production [15]. CIT is converted to arginine in the kidneys after ingestion, which is converted into nitric oxide in the ornithine circuit [16]. Arginine increases nitric oxide, causing vasodilatation, which improves circulation in both central and peripheral blood vessels, and increases growth hormone secretion, which promotes protein anabolism and wound healing [17]. A previous animal study showed that CIT intake increased protein synthesis and protein content in muscles [18]. Another study reported that the intake of both CIT and LEU could stimulate MPS, which resulted from the activation of the mTOR complex 1 (mTORC1) signaling pathway [19].

Therefore, combined intake of CIT and LEU, accompanied by exercise, can be expected to prevent the decrease in body compositions of older adults with low BMI. However, relevant studies have not been reported. This study hypothesized that the simultaneous administration of CIT and LEU accompanied by exercise would increase amino acid concentrations in the plasma and improve body composition and physical activity (PA) compared to placebo with exercise. This study aimed to examine the effect of combined intake of CIT and LEU with multicomponent exercise (WBE and SSE) on body composition, PA, and amino acid concentrations in older Japanese women with low BMI using a randomized, double-blind, placebo-controlled design.

## 2. Materials and Methods

### 2.1. Ethical Approval and Participants

This randomized, double-blind, placebo-controlled study was approved by ethical committees of the University of Tsukuba (reference no. Tai 27-144) and was registered in the University Hospital Medical Information Network center (UMIN no. 000022385). To ensure the reliability of a double-blind trial, the recruitment and data management of the participants were entrusted to a business consignment agency (Tsukuba Health Frontier; THF Co., Ltd., Tsukuba, Japan). Older women residing in Tsukuba City, Japan, were recruited through regional information magazines (Joyo Living Co., Ltd., Tsukuba, Japan) over 1 month. Using G * Power 3.1 analysis was performed to estimate the sample size prior to the start of the study. The power analysis set for an effect size = 0.25, α = 0.05, power (1-β) = 0.8, number of groups = 2, and number of measurements = 3 showed that 28 participants were required for the total sample size. A screening survey was conducted through telephone interview using self-reported, general health questionnaires. The inclusion criteria were: (1) age ranging from 65 to 80 years; (2) BMI ranging from 16 to 21 kg/m^2^ [20]; (3) no exercise prohibition from doctors; and (4) independent mobility and active participation in the exercise classes of the study. Participants were excluded if they (1) used medication for neurological disorders; (2) had a history of comorbid diseases, such as diabetes and brain, liver, kidney, heart, and peripheral vascular diseases; (3) qualify to at least one question of the physical strength section (questions 6 to 10) of the “Kihon Checklist” [21], which is a measure used to identify frailty; (4) had excessive intake of alcohol (>60 g/day) [22]; (5) smoked; (6) had allergies to the supplements administered in this study; (7) had undergone blood collection of more than 400 mL within 10 weeks, or 200 mL within 4 weeks, or had donated blood in the past 2 weeks; and (8) had participated in other clinical studies in the past 10 weeks. A total of 43 older women applied for this study; however, 15 applicants were excluded according to the criteria and two applicants dropped out because of conflicting schedules. Eligible participants were fully informed face-to-face of the study objectives, design, criteria of inclusion and exclusion, intervention of exercise program and supplements, assessments, insurance compensation for injury, withdrawal of consent, and privacy protection. Finally, written consent to participate and for data publishing was obtained from 26 participants.

### 2.2. Study Design

**Figure 1:**
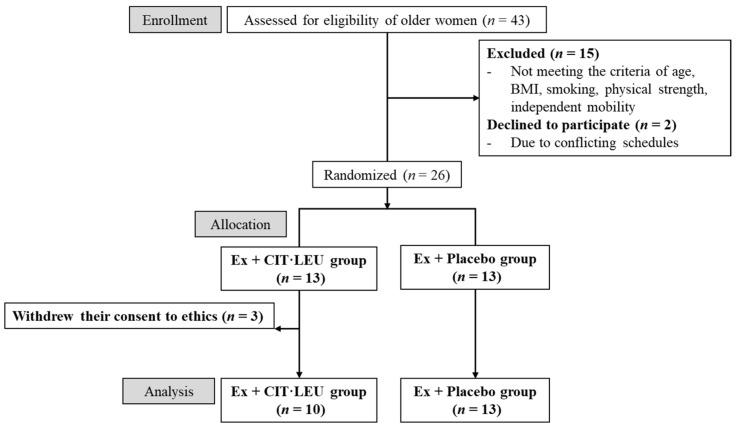
Flow-chart diagram.

THF Co., Ltd. (Tsukuba, Japan) was entrusted to divide 26 participants into two groups using simple randomization method: 13 female participants were assigned to the Ex + CIT·LEU group (exercises and L-CIT and L-LEU) and 13 to the Ex + Placebo group (exercises and placebo). First, random numbers were generated by a computer and assigned to participants. Random numbers lower than 0.5 were determined as A (Ex + CIT·LEU group), and numbers greater than 0.5 were determined as B (Ex + Placebo group). No difference in age and BMI was found between the two groups. However, three participants in the Ex + CIT·LEU group withdrew their consent to participate because of personal reasons. Finally, 23 participants were included in this study (Figure 1). To ensure the reliability of this double-blind study, all data (class attendance records, exercise and food diary, and all measurement results) were entrusted to THF Co., Ltd. (Tsukuba, Japan) The supplements for both the CIT·LEU group and placebo group were prepared to look identical. THF Co., Ltd. (Tsukuba, Japan) distinguished the supplements for the two groups by different numbers marked on top of the package and distributed them to the participants once every 2 weeks. The members of each group remained completely anonymous to both participants and researchers until the key codes were revealed before data analysis. During the 20-week intervention period (July to November 2016), measurements were conducted three times at baseline (Pre: pre-intervention), after 10 weeks (Mid: mid-intervention), and after 20 weeks (Post: post-intervention) in the Innovation Medical Research Institute of the University of Tsukuba.

Within the 20-week intervention period, the two groups equally performed WBE and SSE with the guidance of a professional instructor once a week. Exercise sessions lasted for 75 min (10-min warm-up, 25-min SSE, 30-min WBE, and 10-min cool down). WBE consisted of two training patterns (pattern A, five exercises on the chair, including knee extension, knee raise, squat, heel raise, and abdominal roll-up; pattern B, five exercises on the mat, including pressing a towel between palms, supine bridge, side-lying leg raise, pressing towel between knees, and sit-up), and only one set of WBE was performed with each exercise repeated 10 times. In addition, to reduce the possible burden of this exercise intervention, which lasted for 6 months, the participants were required to practice pattern A or pattern B alternately daily at home. One exercise session of pattern A or pattern B lasted for 25 min (5-min warm-up, 15-min WBE, and 5-min cool down) at home. Then, participants self-evaluated their performance at four levels based on how many repetitions they had completed for each exercise, i.e., 1, certainly (10 times); 2, moderately (≥5 times) 3, slightly (<5 times); 4, could not (0 time). Practice rate of WBE at home was calculated using the percentage of each level. Following this, the daily records were checked, and feedback was provided once every 2 weeks. SSE, a detailed description of which has been introduced in previous studies [11], was practiced on a thin mat measuring 250 cm × 100 cm with 40 squares (25 × 25 cm). SSE contained step patterns of forward, backward, lateral, and oblique movements; in addition, step patterns became increasingly more complex and were categorized into six levels: junior, basic, semiregular, regular, senior, and master. Participants were required to memorize the patterns and then step forward continuously without treading on the front and side lines of squares.

All participants were required to ingest 4.1 g of supplements (Kyowa Hakko Bio Co., Ltd., Tokyo, Japan) with 100 mL of water twice a day (8.2 g) for the duration of the trial. The 16.0 kcal total calorie supplementation in the Ex + CIT·LEU group consisted of 0.8 g CIT, 1.6 g LEU, 0.3 g valine, 0.3 g isoleucine, and 1.1 g carbohydrate. The 16.1 kcal total calorie supplementation in the Ex + Placebo group consisted of only 0.3 g valine, 0.3 g isoleucine, and 3.5 g carbohydrate. These supplements were distributed to all participants once every 2 weeks; supplement intake was confirmed using self-reported diaries. In addition, the participation rate of exercise classes, practice rate of WBE at home, and intake rate of the amino acid supplement were calculated.

### 2.3. Characteristics of the Participants

Systolic and diastolic blood pressure plus heart rate (Omron HEM-7111, Kyoto, Japan) were measured. Participants were required to roll up their sleeves, and the sensor of the blood pressure monitor was placed on the area of the left arm where the brachial artery was located; thereafter, systolic blood pressure, diastolic blood pressure, and heart rate were measured. The unit of blood pressure was millimeters of mercury (mmHg) and that of the heart rate was beats per min (bpm). Additionally, variables used to diagnose sarcopenia (hand grip strength: HGS, 5-m habitual walk) were measured. To measure HGS (T.K.K.5401, Niigata, Japan), participants gripped a dynamometer twice with each hand using maximum force in a standing position, and the average of the maximum force of both hands was used for analysis. In terms of 5-m habitual walk, participants were required to walk through an 11-m straight course at a normal speed, and time from 3-m to 8-m of the course was recorded.

### 2.4. Body Composition

To measure height, participants were required to stand on the height scale with bare feet and look straight. Centimeters were used as the unit of height, and the value was specified to only one decimal place. Body weight was measured in kg with a body composition analyzer (MC-980A, Tanita, Tokyo, Japan). Participants were required to wear light clothes and remove their shoes before measurement. Considering the weight of the clothes, 0.5 kg was subtracted from the obtained body weight and specified to only one decimal place. BMI (kg/m^2^) was calculated as body weight divided by height squared. Dual-energy X-ray absorptiometry (DXA, QDR Discovery Wi, Hologic, Tokyo, Japan) was performed by a clinical technician in a dedicated room, which was quiet and dark. Data regarding body fat, bone area, bone mineral density, lean mass, fat mass, bone mass, and body mass (the sum of the lean mass, fat mass, and bone mass) were obtained at baseline and after the intervention. Participants were required to fast for 10 h in preparation for DXA assessment, wear light clothes, remove their shoes, and lie in a supine position on the DXA machine for 7 min. Appendicular lean mass index (kg/m^2^) was calculated as the sum of lean mass in both arms and legs divided by the square of the height.

### 2.5. Physical Activity

PA was assessed using the Japanese version of the Physical Activity Scale for the Elderly (PASE), the validity and reliability of which have been confirmed by previous studies. The Japanese version of PASE showed an intraclass correlation coefficient 0.65 (0.58–0.72) between the first and second surveys. As for the concurrent validity, the PASE score was significantly correlated with walking steps (ρ = 0.17), energy expenditure (ρ = 0.16) and activity measured with the Japan Arteriosclerosis Longitudinal Study Physical Activity Questionnaire (JALSPAQ) (ρ = 0.48). The PASE is a 12-item questionnaire that measures the average time spent on daily physical activities during the past 7 days. The 12 items are divided in sections on leisure time PA (such as walking, recreational activities, and strength training), household PA (such as home repair and garden maintenance), and occupational PA (such as job and volunteer work). These items are weighted based on the intensity of each activity, and the PASE (total PA) score is the sum of the 12 weighted items [23].

### 2.6. Amino Acid Concentrations

Analysis of blood samples was entrusted to a microbiological institute (Kotobiken Medical Laboratories Inc., Tsukuba, Japan). Participants were instructed to fast for 10 h in preparation for venipuncture of the brachial veins. Blood was collected using EDTA-2NA containing tubes by nurses. To separate the plasma, 2 mL of blood was collected and centrifuged at 3000 rpm for 10 min at 4 °C, and 0.5 mL of plasma was stored at −80 °C until analysis. Amino acid concentrations were analyzed using high-performance liquid chromatography with an acquity ultra performance liquid chromatography mass spectrometer system with a mass trak AAA amino acid analysis solution kit (Waters Co., Ltd., Tokyo, Japan) [24].

### 2.7. Statistical Analysis

The primary outcome was change in body composition, and secondary outcomes were changes in PA and amino acid concentrations. The Mann–Whitney U test was used to compare the quantitative variables between the two groups at baseline, and two-way repeated measures analysis of variance was used to evaluate differences in the effects of the intervention on body composition, PA, and amino acid concentrations. Covariates included height, body weight, and baseline values of each variable. Excluding height, body weight, and BMI, other components of body composition were adjusted for height, body weight, and their baseline values. Moreover, PA and amino acid concentrations were adjusted for their baseline values. Based on significant interactions (groups × times), this study conducted post hoc analyses with Bonferroni correction. To verify the effect sizes of all variables from Pre to Post, Cohen’s d value was calculated as the difference between two means divided by a pooled standard deviation (small, d = 0.2; medium, d = 0.5; large, d = 0.8) [25]. All statistical analyses were performed using SPSS version 26.0 (IBM Corp., Armonk, NY, USA), with the level of significance set at *p* < 0.05.

## 3. Results

### 3.1. Characteristics of Participants

Characteristics of participants at baseline are shown in Table 1. Significant differences in body weight (*p* = 0.03) and systolic blood pressure (*p* = 0.01) were observed between the two groups. No significant differences were found in other variables. In addition, the intake rate of amino acid supplementation and the participation rate of exercise classes of both groups were >90%.

### 3.2. Body Compositions

In Table 2 and Table 3, the unadjusted model showed significant interactions in both body weight and BMI. According to the post hoc analysis, body weight and BMI showed greater increase at Post when compared with their values at both Pre and Mid in Ex + CIT·LEU group. In the adjusted model, significant interactions were observed in lean mass (*p* = 0.04) and body mass (*p* = 0.02). According to the post hoc analysis, body mass showed a significant increase from Pre to Post in the Ex + CIT·LEU group. Despite that effect sizes of body weight (d = 0.4), BMI (d = 0.3), body mass (d = 0.3), and lean mass (d = 0.2) were small in the Ex + CIT LEU group, the values were larger than those in Ex + placebo group.

### 3.3. Physical Activity

In Table 4, the unadjusted model showed significant interactions in household PA (*p* = 0.04) and total PA (*p* = 0.04). According to the post hoc analysis, the increase between Mid and Post was greater in the Ex + CIT·LEU group. Large effect sizes were observed in leisure time PA (Ex + CIT·LEU group; d = 1.1, Ex + Placebo group; d = 1.4), household PA (Ex + CIT·LEU group; d = 1.6), and total PA (Ex + CIT·LEU group; d = 1.5).

### 3.4. Amino Acid Concentrations in Plasma

Table 5 shows the amino acid concentrations in plasma. In both the unadjusted model and adjusted model, interactions with tyrosine (*p* = 0.02, 0.03) and phenylalanine (*p* = 0.04, 0.03) were significant. According to the post hoc analysis, a more significant increase in tyrosine at Post was observed compared with that at Pre in both groups; however, the increase in phenylalanine was greater in the Ex + CIT·LEU group. In both groups, large effect sizes can be observed in arginine (Ex + CIT·LEU group; d = 1.4, Ex + Placebo group; d = 1.3), glutamic acid (Ex + CIT·LEU group; d = 0.8, Ex + Placebo group; d = 1.0), and methionine (Ex + CIT·LEU group; d = 1.4, Ex + Placebo group; d = 0.8). In the Ex + CIT·LEU group, large effect sizes can be observed in glutamine (d = 0.8), alanine (d = 0.8), and tyrosine (d = 1.1). In the Ex + Placebo group, large effect sizes can be observed in glycine (d = 0.9), cystine (d = 0.9), and lysine (d = 0.9).

## 4. Discussion

Inevitable decrease in body composition induced by aging presents a challenge to physicians, and it has been reported that muscle mass decreases by 12.9% in men and 5.3% in women after 10 years, and body weight decreases by 9.9–19.4 kg [26]. The health aging body composition study (health ABC study), a 5-year longitudinal study, reported that muscle mass increased with gain in body weight, whereas fat mass increased regardless of change in body weight [27]. Results in this study were consistent with those of the health ABC study [27] showing that an increase in body weight was strongly associated with an increase in muscle mass; however, different results were observed in changes in fat mass. This may be due to the lower prevalence of obesity among Asians than among Americans.The participants’ fat mass at baseline (Ex + CIT·LEU: 10.7 kg, Ex + Placebo: 12.3 kg, Table 3) in this study was lower than those in the Health ABC study (28.2 kg).

Results of this study indicated that CIT and LEU intake combined with exercises could promote improvement on body weight and lean mass. Significant larger increase in body mass was found in the CIT·LEU group. Despite the fact that a non-significant difference between groups was found in lean mass through Bonferroni analysis, significant interaction between groups and time was observed and the effect size in lean mass of the CIT·LEU group was obviously larger than that of the Placebo group. CIT has been reported to promote MPS by stimulating the mTORC1 signaling pathway in rats [19]. Additionally, old rats ingesting both CIT and LEU have shown higher protein content in muscle mass [18]. Therefore, combined intake of CIT and LEU stimulates protein synthesis to a greater extent than LEU intake alone, and the results of the present study also demonstrated the beneficial effect of the combined intake of CIT and LEU on older adults. WBE can be easily performed in daily life without tools and weight burden and is a particularly useful exercise prescription for older adults. In addition, WBE mobilizes multiple muscles exerting weight on joints and stimulates bone metabolism by creating new bone cells against gravity. As WBE can effectively improve muscle strength and joint mobility, it can be used to prevent geriatric syndromes, such as sarcopenia and osteopenia [9,10]. According to a meta-regression analysis study, muscle mass, muscle strength, and physical function in older adults with frailty or sarcopenia were improved significantly by combined intervention of muscle strengthening exercise (MSE) and protein supplementation (PS) compared to MSE or PS alone [28]. Therefore, the present study supports the results of previous studies, indicating that a combined intervention of exercise (WBE + SSE) and nutrition (CIT + LEU) can effectively increase body weight and lean mass in older adults.

A prospective cohort study in Japan reported that both low BMI and high BMI (obesity) were strongly associated with mortality, showing a U-shaped curve [29]. High BMI and low PA are known to have a relationship [30]; however, the relationship between low BMI and low PA has not been reported previously. Moreover, low BMI may positively relate to low PA because low BMI is a risk factor for sarcopenia, while a decreased PA is often a consequence of sarcopenia; therefore, the presence of sarcopenia may indirectly affect the relationship between the two variables [31]. According to a systematic review and meta-analysis, decrease in PA may increase the incidence of sarcopenia (odds ratio = 0.45; 95% confidence interval 0.37–0.55) [32], suggesting a potentially complex bidirectional relationship between the two variables. In the present study, the household PA and total PA of older women with low BMI increased significantly in the Ex + CIT·LEU group. A previous study has reported that, compared to Placebo, supplementation containing whey protein (22 g), essential amino acids (10.9 g, including leucine (4 g), and vitamin D was effective to improve fat-free mass, physical function, as well as activities of daily living in the sarcopenic elderly [33]. Moreover, another previous study reported that, after 24 weeks of intervention, total PA improved significantly and was maintained in the resistance exercise and nutrition (mainly 8.61 g protein, etc.) group compared with the control group. Moreover, lower limb muscle mass and appendicular skeletal muscle mass were also increased [34], which is consistent with the findings in this study. These results suggest that exercise and protein intake can improve both body composition and daily PA. The risk factors of sarcopenia include being underweight [4], being female sex, and having low BMI, low PA, and poor mobility [31]. Therefore, the combination of exercise and nutrition intervention is more effective for older women with low BMI to prevent sarcopenia.

In general, protein from food is hydrolyzed into peptides and amino acids and then absorbed by muscles and organs through blood circulation. Considering the constant amount of amino acid transporters, amino acids ingested may fail to be fully absorbed due to their saturation concentrations. However, compared to intact protein, free-form essential amino acid (EAA) requires no digestion and can be absorbed rapidly. Furthermore, EAA may be more effective to increase the total EAA concentration and stimulate MPS than intact protein and whole-food protein and mixed meals. In addition, combined ingestion of two or more whole-food proteins has been reported to optimize EAA intake, and MPS was stimulated by increase of isolated intact proteins with EAA over a 1–3 h postprandial period. It was also reported that protein intake after exercise contributes to the sustained stimulation of MPS. Moreover, the effect of EAA, intact protein, and whole-food protein on MPS depends on exercise and lifestyle in a dose-dependent manner [35]. Combined interventions of exercise and LEU-rich EAA for 3 months have been reported to increase leg muscle mass in Japanese women with sarcopenia [8]. In that study, they defined sarcopenia as BMI ≤ 22 kg/m^2^ and found that exercise improved the muscle mass of older women with low BMI, which is consistent with our findings. In another study, supplement intake (6 g CIT maleate, 5 g creatine, 3 g LEU, 3 g isoleucine, 1.5 g valine, and others) and resistance exercise for 4 weeks significantly increased body mass and lean body mass compared with the placebo group (digestion-resistant maltodextrin) [36]. The participants of the previous study were recreationally active men, whereas this study focused on older women with low BMI; however, the effects of CIT and LEU intake and exercise were consistent between the studies. However, other studies have reported conflicting results. A previous study reported that a high dose of whey protein (45 g) intake, rather than combined intake of low dose whey protein (15 g) and CIT (10 g), promoted postprandial MPS after resistance exercise in older men [37]. Another study reported that CIT intake (0.18 g/kg/day) increased plasma CIT and promoted arginine availability but exerted no influence on LEU oxidation and whole-body protein synthesis [38]. This finding contradicted that of a previous study reporting that CIT intake increased arginine availability in the urea cycle, which promotes protein anabolism, indicating that CIT may affect protein synthesis [18,19]. These conflicting findings may have resulted from the various doses and durations of CIT and LEU intake in each study. According to a review, the effective dose for daily CIT supplementation was recommended at a minimum of 3 g to a maximum of 10 g [39]. The dose of CIT administered in this study was lower than the one reported in that review. In addition, the World Health Organization recommends doses for daily intake of LEU (0.039 g/kg), isoleucine (0.020 g/kg), and valine (0.026 g/kg) [40]. This study showed that LEU at a dose of 0.072 g per body weight (kg), calculated according to the average body weight of all participants (44.1 kg) and the daily intake of LEU (3.2 g), was effective compared with previous studies.

Considering caloric balance, uniform doses of valine (0.6 g) and isoleucine (0.6 g) were administered to both groups, and thus their intake in the Ex + Placebo group may have influenced the results. Therefore, administration of only carbohydrates to the Ex + Placebo group should be considered in future studies. Although the concentrations of CIT and LEU in plasma showed no significant interaction between the two groups, a tendency of interaction (*p* = 0.07) was observed in the CIT level of the unadjusted model and the LEU level of the adjusted model. In addition, the effect size was larger in the Ex + CIT·LEU group (CIT: d = 0.7, LEU: d = 0.5) than in the Ex + Placebo (CIT: d = 0.2, LEU: d = 0.1) group, and the intake rate of the amino acid supplement in the Ex + CIT·LEU group was 96.5%. These results suggest that CIT and LEU supplements were well received by the participants, and a long-term intake of CIT and LEU effectively increased their plasma levels. However, the amount of leucine in plasma (113.5 nmol/mL) may be insufficient to impact MPS robustly in older adults in this study. In addition, the concentration of tyrosine significantly increased in both groups, but the effect size was larger in the Ex + CIT·LEU group (d = 1.1) than in the Ex + Placebo group (d = 0.5). The concentration of phenylalanine increased significantly only in the Ex + CIT·LEU group. Phenylalanine is an essential amino acid converted to tyrosine during protein metabolism, which plays a vital role in transmitting signals between the brain and nerve cells. After a mixed diet (49% carbohydrate, 22% protein, 29% lipid), tracers of both leucine and phenylalanine-tyrosine increased in the plasma and intracellular precursor pools, which indicates increased whole-body protein synthesis [41]. With reference to previous studies, LEU intake may have increased the phenylalanine and tyrosine concentrations in this study; however, this requires further research.

This study has several limitations. First, there was sampling bias and a relatively small sample size. Forty-three older women living in rural Japan were recruited, but 20 participants dropped out because of the exclusion criteria and personal reasons. Therefore, further studies with a larger population that adheres to the same intervention design is necessary. Second, daily diet containing CIT and LEU was not controlled in this study, and differences across individual lifestyles (diet, sleep, exercise, etc.) may have also influenced the amino acid concentrations in the plasma. This may have led to the non-significant interaction between the two groups; therefore, it is necessary to control daily diet and conduct a survey on lifestyle in future studies. Third, PA was measured using the PASE questionnaire, which is a reliable tool to evaluate different types of PA in the elderly [23]. However, reporting bias may exist, and other methods to obtain objective data, such as use of an accelerometer, should be considered in future studies.

## 5. Conclusions

This study demonstrated that body weight, BMI, body mass, household PA, total PA, tyrosine, and phenylalanine significantly increased after 20 weeks in the Ex + CIT·LEU group. This study suggests that the combined intake of CIT and LEU accompanied by multicomponent exercise can improve body weight, BMI, body mass, and PA in older women with low BMI, contributing to the prevention of sarcopenia and frailty. However, to better illustrate the outcomes of this study, additional examination to clarify the influence from daily energy, protein and specific amino acid intake seems necessary in further researches.

## Figures and Tables

**Table 1:** Characteristics of the participants on baseline.

		Ex + CIT·LEU (*n* = 10)			Ex + Placebo (*n* = 13)			
Variables	Unit	Mean	±	SD	Mean	±	SD	*p*-Value
Age	(year)	69.5	±	3.7	71.2	±	5.8	0.69
Height	(cm)	151.1	±	4.0	155.4	±	5.1	0.09
Body weight	(kg)	42.4	±	2.4	45.7	±	3.5	0.03
BMI	(kg/m^2^)	18.6	±	1.5	18.9	±	1.1	0.56
Systolic blood pressure	(mmHg)	119.1	±	12.2	132.5	±	13.3	0.01
Diastolic blood pressure	(mmHg)	68.0	±	7.0	72.9	±	10.4	0.17
Heart rate	(bpm)	75.8	±	5.8	79.8	±	11.0	0.17
Intake rate of amino acid supplementation	(%)	96.5	±	5.0	96.9	±	5.0	0.69
Participation rate of exercise classes	(%)	96.1	±	5.3	91.5	±	9.2	0.23
Hand grip strength	(kg)	21.0	±	4.4	22.7	±	3.0	0.66
5-m habitual walk	(sec)	4.1	±	0.4	4.1	±	1.0	0.39
Appendicular lean mass index	(kg/m^2^)	5.0	±	0.4	5.2	±	0.5	0.54

Note: Mann–Whitney U test, *p* < 0.05, *n*: number of participants, SD: standard deviation, Ex: exercise, CIT: citrulline, LEU: leucine, BMI: body mass index.

**Table 2:** Effects of amino acids (CIT and LEU) intake and multicomponent exercises on body compositions (Variables measured in Pre, Mid and Post).

			Ex + CIT·LEU (*n* = 10)			Ex + Placebo (*n* = 13)			Main Effect of Time *p*-Value (Pre-Mid-Post)	Interaction *p*-Value (Groups × Times)	Post Hoc Analysis with Bonferroni Correction	^†^ Main Effect of Time *p*-Value (Pre-Mid-Post)	^†^ Interaction *p*-Value (Groups × Times)	^†^ Post Hoc Analysis with Bonferroni Correction
Variables	Unit	Time	Mean	±	SD	Mean	±	SD						
Height	(cm)	Pre	151.1	±	4.0	155.4	±	5.1	0.01	0.78				
		Mid	150.9	±	3.8	155.1	±	5.1						
		Post	151.1	±	3.9	155.4	±	5.2						
	Effect size		0			0								
Body weight	(kg)	Pre	42.4	±	2.4	45.7	±	3.5	0.05	0.03	Ex + CIT·LEU: Pre, Mid< Post			
		Mid	42.8	±	3.1	45.7	±	3.6						
		Post	43.4	±	3.2	45.6	±	3.6						
	Effect size		0.4			0								
BMI	(kg/m^2^)	Pre	18.6	±	1.5	18.9	±	1.1	0.04	0.01	Ex + CIT·LEU: Pre, Mid < Post			
		Mid	18.9	±	1.7	19.0	±	1.0						
		Post	19.1	±	1.6	18.9	±	1.1						
	Effect size		0.3			0								

Note: Two-way repeated measures analysis of variance, *p* < 0.05, CIT: citrulline, LEU: leucine, BMI: body mass index, Effect size (Cohen’s d): Pre vs. Post, 0.2: small, 0.5: medium, 0.8: large, ^†^: Adjusted for height, body weight, and baseline values of each variable.

**Table 3:** Effects of amino acids (CIT and LEU) intake and multicomponent exercises on body compositions (Variables measured in Pre and Post).

Variables	Unit	Time	Ex + CIT·LEU (*n* = 10)			Ex + Placebo (*n* = 13)			Main Effect of Time *p*-Value (Pre-Post)	Interaction *p*-Value (Groups × Times)	Post Hoc Analysis with Bonferroni Correction	^†^ Main Effect of Time *p*-Value (Pre-Post)	^†^ Interaction *p*-Value (Groups × Times)	^†^ Post Hoc Analysis with Bonferroni Correction
			Mean	±	SD	Mean	±	SD						
Body fat	(%)	Pre	25.6	±	6.5	27.7	±	4.3	0.28	0.23		0.84	0.53	
		Post	26.3	±	6.5	27.7	±	4.0						
	Effect size		0.1			0								
Bone area	(cm^2^)	Pre	1500.2	±	125.2	1617.5	±	131.8	0.89	0.12		0.26	0.17	
		Post	1508.5	±	120.1	1609.8	±	131.2						
	Effect size		0.1			0.1								
Bone mineral density	(g/cm^2^)	Pre	0.99	±	0.13	0.98	±	0.09	0.01	0.49		0.38	0.88	
		Post	0.99	±	0.12	0.97	±	0.09						
	Effect size		0.1			0.1								
Bone mass	(g)	Pre	1498.1	±	277.3	1594.5	±	264.2	0.02	0.11		0.76	0.36	
		Post	1494.4	±	264.0	1565.8	±	253.2						
	Effect size		0			0.1								
Fat mass	(kg)	Pre	10.7	±	3.3	12.3	±	2.5	0.30	0.09		0.97	0.22	
		Post	11.2	±	3.5	12.3	±	2.3						
	Effect size		0.1			0								
Lean mass	(kg)	Pre	29.1	±	1.3	30.4	±	2.2	0.79	0.30		0.12	0.04	N.S
		Post	29.4	±	1.3	30.3	±	2.4						
	Effect size		0.2			0								
Body mass	(kg)	Pre	41.3	±	2.6	44.3	±	3.4	0.41	0.06		0.92	0.02	Ex + CIT·LEU: Pre < Post
		Post	42.1	±	3.2	44.1	±	3.5						
	Effect size		0.3			0.1								

Note: Two-way repeated measures analysis of variance, *p* < 0.05, CIT: citrulline, LEU: leucine, Body mass: sum of the bone mass, fat mass and lean mass, N.S: not significant, Effect size (Cohen’s d): Pre vs. Post, 0.2: small, 0.5: medium, 0.8: large, ^†^: Adjusted for height, body weight, and baseline values of each variable.

**Table 4:** Effects of amino acids (CIT and LEU) intake and multicomponent exercises on physical activity.

Variables	Unit	Time	Ex + CIT·LEU (*n* = 10)			Ex + Placebo (*n* = 13)			Main Effect of Time *p*-Value (Pre-Mid-Post)	Interaction *p*-Value (Groups × Times)	Post Hoc Analysis with Bonferroni Correction	^†^ Main Effect of Time *p*-Value Pre-Mid-Post)	^†^ Interaction *p*-Value (Groups × Times)
			Mean	±	SD	Mean	±	SD					
Leisure time PA	(score)	Pre	14.9	±	13.2	13.3	±	9.0	<0.01	0.81		<0.01	0.84
		Mid	38.1	±	27.1	33.6	±	15.5					
		Post	32.3	±	17.0	28.1	±	12.3					
	Effect size		1.1			1.4							
Household PA	(score)	Pre	52.0	±	14.6	87.5	±	35.6	0.02	0.04	Ex + CIT·LEU: Pre < Mid, Post	<0.01	0.25
		Mid	77.4	±	24.2	82.5	±	27.6					
		Post	80.7	±	20.5	93.4	±	23.6					
	Effect size		1.6			0.2							
Occupational PA	(score)	Pre	9.3	±	18.8	6.6	±	20.0	0.44	0.92		0.76	0.75
		Mid	9.3	±	17.9	6.5	±	19.9					
		Post	8.1	±	17.9	4.8	±	13.6					
	Effect size		0.1			0.1							
Total PA	(score)	Pre	76.2	±	31.8	107.4	±	50.0	<0.01	0.04	Ex + CIT·LEU: Pre < Mid, Post	<0.01	0.15
		Mid	124.8	±	46.0	122.5	±	35.3					
		Post	121.1	±	29.6	126.3	±	32.5					
	Effect size		1.5			0.5							

Note: Two-way repeated measures analysis of variance, *p* < 0.05, CIT: citrulline, LEU: leucine, PA: Physical activity, Effect size (Cohen’s d): Pre vs. Post, 0.2: small, 0.5: medium, 0.8: large, ^†^: Adjusted for baseline value of each variable.

**Table 5:** Effects of amino acids (CIT and LEU) intake and multicomponent exercises on amino acid concentrations.

Variables	Unit	Time	Ex + CIT·LEU (*n* = 10)			Ex + Placebo (*n* = 13)			Main Effect of Time *p*-Value (Pre - Post)	Interaction *p*-Value (Groups × Times)	Post Hoc Analysis with Bonferroni Correction	^†^ Main Effect of Time *p*-Value (Pre - Post)	^†^ Interaction *p*-Value (Groups × Times)	^†^ Post Hoc Analysis with Bonferroni Correction
			Mean	±	SD	Mean	±	SD						
Arginine	(nmol/mL)	Pre	69.3	±	14.2	65.2	±	16.0	<0.01	0.68		<0.01	0.37	
		Post	92.5	±	18.3	85.1	±	13.9						
	Effect size		1.4			1.3								
Citrulline	(nmol/mL)	Pre	32.4	±	6.8	34.5	±	6.9	<0.01	0.07		0.03	0.11	
		Post	37.1	±	7.6	36.0	±	6.2						
	Effect size		0.7			0.2								
Leucine	(nmol/mL)	Pre	104.0	±	23.5	105.7	±	17.8	0.05	0.12		<0.01	0.07	
		Post	113.5	±	19.0	107.3	±	13.1						
	Effect size		0.5			0.1								
Valine	(nmol/mL)	Pre	195.7	±	35.8	197.8	±	31.1	<0.01	0.79		0.03	0.82	
		Post	219.1	±	35.6	218.2	±	33.0						
	Effect size		0.7			0.6								
Isoleucine	(nmol/mL)	Pre	49.2	±	11.2	52.6	±	9.3	<0.01	0.25		0.01	0.37	
		Post	56.4	±	11.7	56.3	±	8.3						
	Effect size		0.6			0.4								
Threonine	(nmol/mL)	Pre	114.7	±	14.8	110.4	±	19.9	<0.01	0.52		0.28	0.58	
		Post	125.3	±	20.2	126.3	±	27.3						
	Effect size		0.6			0.7								
Serine	(nmol/mL)	Pre	125.2	±	14.0	116.0	±	14.0	0.08	0.23		0.30	0.33	
		Post	126.4	±	20.7	124.2	±	16.1						
	Effect size		0.1			0.5								
Asparagine	(nmol/mL)	Pre	59.8	±	7.8	59.8	±	8.9	0.06	0.36		0.27	0.36	
		Post	59.0	±	10.6	57.1	±	10.1						
	Effect size		0.1			0.3								
Glutamic acid	(nmol/mL)	Pre	34.3	±	7.1	35.4	±	5.3	<0.01	0.31		0.80	0.35	
		Post	41.6	±	10.1	48.1	±	17.2						
	Effect size		0.8			1.0								
Glutamine	(nmol/mL)	Pre	636.6	±	58.9	632.7	±	75.4	<0.01	0.81		<0.01	0.83	
		Post	690.5	±	74.1	679.3	±	56.2						
	Effect size		0.8			0.7								
Glycine	(nmol/mL)	Pre	230.5	±	43.7	241.6	±	59.1	<0.01	0.08		0.54	0.10	
		Post	259.8	±	58.6	303.2	±	77.3						
	Effect size		0.6			0.9								
Alanine	(nmol/mL)	Pre	313.7	±	43.1	319.6	±	75.1	<0.01	0.84		0.15	0.87	
		Post	364.0	±	83.1	364.7	±	75.3						
	Effect size		0.8			0.6								
Cystine	(nmol/mL)	Pre	50.1	±	8.1	48.4	±	7.2	<0.01	0.13		<0.01	0.15	
		Post	52.4	±	4.6	55.4	±	8.5						
	Effect size		0.3			0.9								
Methionine	(nmol/mL)	Pre	22.8	±	2.2	23.1	±	3.2	<0.01	0.24		0.11	0.27	
		Post	26.8	±	3.5	25.7	±	3.5						
	Effect size		1.4			0.8								
Tyrosine	(nmol/mL)	Pre	57.0	±	11.5	59.6	±	12.5	<0.01	0.02	Ex + CIT·LEU, Ex + Placebo: Pre < Post	0.01	0.03	Ex + CIT·LEU, Ex + Placebo: Pre < Post
		Post	70.5	±	12.6	65.6	±	11.3						
	Effect size		1.1			0.5								
Phenylalanine	(nmol/mL)	Pre	58.2	±	7.6	58.9	±	6.2	0.43	0.04	Ex + CIT·LEU: Pre < Post	0.02	0.03	Ex + CIT·LEU: Pre < Post
		Post	62.2	±	7.5	57.6	±	5.8						
	Effect size		0.5			0.2								
Histidine	(nmol/mL)	Pre	85.0	±	7.2	83.7	±	7.7	<0.01	0.19		0.37	0.18	
		Post	91.6	±	11.0	87.1	±	6.3						
	Effect size		0.7			0.5								
Lysine	(nmol/mL)	Pre	199.5	±	12.6	188.0	±	18.7	<0.01	0.25		0.08	0.47	
		Post	210.2	±	18.0	208.4	±	24.6						
	Effect size		0.7			0.9								
Tryptophan	(nmol/mL)	Pre	45.8	±	7.7	46.5	±	6.1	0.11	0.13		<0.01	0.06	
		Post	50.1	±	3.8	46.8	±	5.6						
	Effect size		0.7			0.1								

Note: Two-way repeated measures analysis of variance, *p* < 0.05, CIT: citrulline, LEU: leucine, Effect size (Cohen’s d): Pre vs. Post, 0.2: small, 0.5: medium, 0.8: large, ^†^: Adjusted for baseline value of each variable.

## Data Availability

All data generated or analyzed during this study are included in this published article. In addition, upon reasonable request, data supporting the findings of the study are provided by the corresponding author.
